# Superior memorizers employ different neural networks for encoding and recall

**DOI:** 10.3389/fnsys.2015.00128

**Published:** 2015-09-14

**Authors:** Johannes Mallow, Johannes Bernarding, Michael Luchtmann, Anja Bethmann, André Brechmann

**Affiliations:** ^1^Institute for Biometry and Medical Informatics, Medical Faculty, Otto-von-Guericke UniversityMagdeburg, Germany; ^2^Center for Behavioural Brain Sciences, Otto-von-Guericke UniversityMagdeburg, Germany; ^3^University Clinic for Neurosurgery, Otto-von-Guericke UniversityMagdeburg, Germany; ^4^Special-Lab Non-Invasive Brain Imaging, Leibniz Institute for NeurobiologyMagdeburg, Germany

**Keywords:** superior memorizers, method of loci, mental navigation, encoding, recall

## Abstract

Superior memorizers often employ the method of loci (MoL) to memorize large amounts of information. The MoL, known since ancient times, relies on a complex process where information to be memorized is bound to landmarks along mental routes in a previously memorized environment. However, functional magnetic resonance imaging data on groups of trained superior memorizer are rare. Based on the memorizing strategy reported by superior memorizers, we developed a scheme of the processes successively employed during memorizing and recalling digits and relate these to brain activation that is specific for the encoding and recall period. In the examined superior memorizers several regions, suggested to be involved in mental navigation and digit-to-word processing, were specifically activated during encoding: bilateral early visual cortex, retrosplenial cortex, left parahippocampus, left visual cortex, and left superior parietal cortex. Although the scheme suggests that some steps during encoding and recall seem to be analog, none of the encoding areas were specifically activated during the recall. Instead, we found strong activation in left anterior superior temporal gyrus, which we relate to recalling the sequential order of the digits, and right motor cortex that may be related to reciting the digits.

## Introduction

In the last two decades, functional magnetic resonance imaging (fMRI), with its capability to non-invasively localize activated brain areas, led to many studies investigating different aspects of memory-related processes. Although neuroimaging studies on encoding and retrieval have focused on prefrontal cortex (PFC) and the medial temporal lobe (MTL) memory system (Scoville and Milner, 1957; Squire and Zola-Morgan, 1991), several other regions have been found to play important roles as well (Kim, 2011). For one, varying paradigms were used to examine episodic memory, such as successful encoding, successful retrieval, and objective and subjective recollection. A major meta-analysis of the results, with a focus on the use of pictures, words, or faces as memoranda, was published by Spaniol et al. (2009). Furthermore, although enhancing memorization capabilities has always received attention, mind-enhancing techniques appear to be gaining increasing interest. This may be partially due to efforts to counteract a decline in memory capacities of elderly persons, especially those suffering from Alzheimer disease. However, it remains debatable whether training strategies can have a long-lasting ameliorating effect on memory capabilities. While emotional episodic memories tend to be easy to retain over long periods, abstract data such as numbers, long lists of words, or chemical formulas are usually harder to keep in mind. However, it is well known that trained memory experts [“superior memorizers” (SMs)] can achieve extraordinary results when memorizing long lists of numbers, sequences of images, or other data in a short time. In this study we wanted to compare the brain areas *specifically* activated by SMs using the method of loci (MoL) on digits during encoding with those activated during recall.

The essence of their strategy consists in visualizing simple or abstract data, such as numbers, as concrete objects and subsequently placing them on landmarks along an internal route that was previously memorized. Those landmarks can be actual objects, like a chair or a tree, but also the empty space between two buildings. The far most popular version of this is the MoL (*locus* means *place* in Latin), a technique from ancient art of mnemonics (Yates, 1966). The transformation of the digits into visual objects can be done by using a *phonetic system* (Worthen, 2010). For recall, the internal route is walked through mentally and the objects transformed back into the original data.

Up to now, only one study analyzed the brain networks employed in SMs when encoding data (Maguire et al., 2003) and one studied subjects who received short-term training in the MoL (Kondo et al., 2005). Furthermore, there are only two other studies that investigated how SMs encode and recite digits. Both studies were performed with only one subject (Hu et al., 2009; Raz et al., 2009). However, applying this method to abstract data such as digits is a very complex, poorly described process. For that reason we developed a detailed scheme of the sub-processes of memorizing and recalling digits by using introspective information from superior memorizers. We then used fMRI to identify and compare the brain areas that are selectively activated by trained memorizers when encoding and recalling a sequence of digits while applying a phonetic system and the MoL. Moreover, we present some hypotheses whether the activated regions might be connected to the presumably underlying internal processing chains, which were described in the detailed scheme.

We suggest that the memorizing of digits, while using the MoL in combination with a phonetic system, not only encompasses visuo-spatial and memory-related components, but also recruits several language-related processes such as word generation and internal speech. Thus, in addition to speech- and language-related visuo-spatial areas, auditory brain networks may be activated. We expected to replicate the findings of Maguire et al. (2003), who described brain areas that are active in SMs when encoding items (including digits). However, Maguire et al. (2003) did not analyze the brain areas that were active during the recall phase. Consequently, we did not have a specific hypothesis, except that, since successful retrieval in normal subjects is suggested to be accompanied by processes similar to those involved in encoding, one could expected at least some overlap of brain areas specifically active during recall and encoding (Rugg et al., 2008).

### Scheme for Encoding and Recall

**FIGURE 1:**
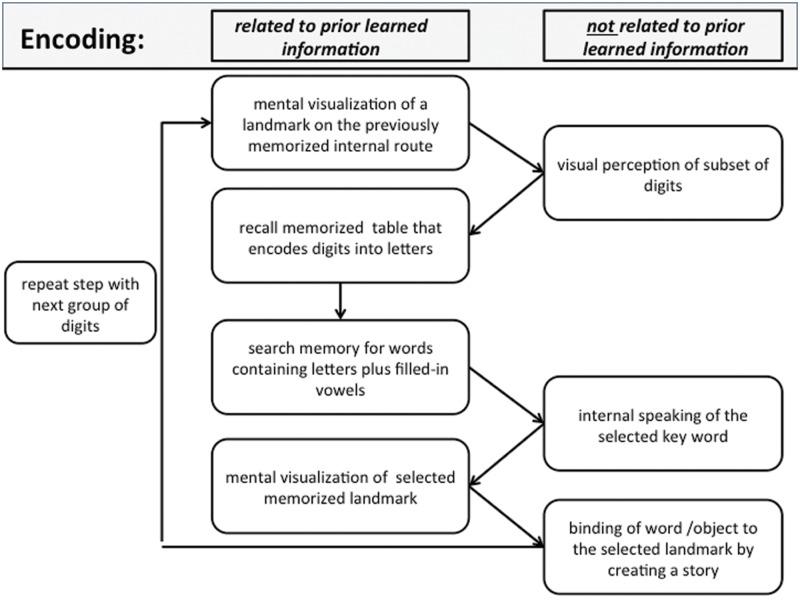
**Encoding: schematic view of a typical processing chain when encoding a sequence of digits using the method of loci (MoL).** See text for further details.

According to the first author, who is one of the best trained superior memorizer in the world and expert on the practical use of the mnemonic methods since more than 10 year, during encoding, SMs usually perform an intricate chain of nested language-related, visual-related, and spatial navigation-related processes. Before encoding, SMs choose a previously learned internal route that they are familiar with. The actual encoding process is then a repetition of the process shown in (**Figure 1**). To emphasize which intermediate steps of the process are related to previously learned information, we divided the process into two columns. The encoding process starts with mental visualization of the first landmark from the chosen route. Then, a subset of items (here two or three digits, individual to each superior memorizer) is transformed into letters using a previously learned digit–letter encoding table. Subsequently, these letters are used to generate key words according to previously learned rules. Usually the key words encode objects that can easily be visualized internally (e.g., the digit sequence “5 0 4” corresponds to “lsr” which is expanded to “laser”). A similar approach is described in (Yin et al., 2015). Finally, the imagined objects are placed on the particular landmark along the internal route. To increase efficiency, the connections between the landmarks and placed items are composed into a small story, often within an emotional context. This process is repeated until all numbers are memorized (**Figure 1**). The detailed scheme in **Figure 1** separates the sub-processes reported and confirmed by SMs when initially learning to encode digits using a phonetic system and the MoL. Note that—depending on the performance level and experience of each superior memorizer—the mental effort differs between subjects for the sub-processes involved in translating the digits into words (e.g., recall of the letter table, search for words, and silent speaking of the word). This reportedly requires less effort for experienced memorizers than for naïve subjects or when learning the mnemonic strategies.

**FIGURE 2:**
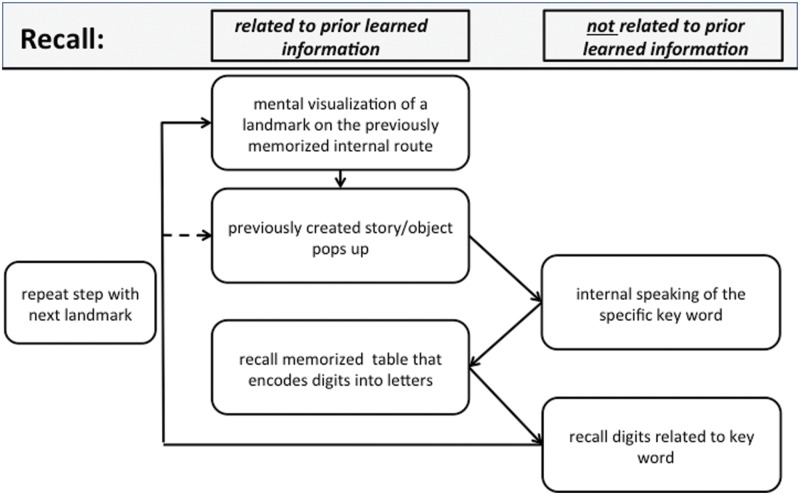
**Recall: schematic view of a typical processing chain when recalling a sequence of digits using the MoL.** See text for further details.

Similar to the encoding process, recall (**Figure 2**) begins by mentally visualizing the first landmark from the used route. Now the previously created story or object pops into mind and the memorizer decodes the related key word, using the digit–letter table. After recalling this subset of digits, the memorizer moves on to the next landmark.

According to the first author, the described method seems to be used by almost everybody in the world of ranked memory championships. Anyway there may be other memory experts who uses different approaches, like shown by Richman et al. (1995).

## Materials and Methods

### Subjects

Eleven SMs (eight male, three female; mean age 30.8 years) and eleven control subjects (six male, five female; mean age 26.3 years) participated in the study. All of the SMs were highly trained in the MoL and have proven their abilities in public competitions of memory performance. They all had perfectly memorized at least 100 digits in 5 min and thus belong to a very exclusive group of SMs worldwide. To give an impression about the group size, it should be mentioned that at the time of the experiment only about 200 people worldwide had proved that capability. The performance variability for the subjects in that study was between 100 and 320 perfectly memorized digits in 5 min. All subjects confirmed to use the strategy, including the digit–letter transformation and the MoL, as shown in **Figure 1**. The reported time of experience with the methods was between 3 and 8 years. None of the control subjects reported having experience in the MoL or any other mnemonic technique and they where not provided with those strategies or any other method. The data from four SMs and four control subjects had to be excluded from the fMRI analysis due to excessive head motion (>2 mm and/or 2°). This large number of participants that has to be excluded is possibly due to the overt speech response required during the recall and rest condition. The mean age was 32.3 years for the remaining seven SMs (five male, two female, and one left-hander) and 25.1 years for the seven control subjects (four male, three female, and all right-handed). All subjects gave their written informed consent to the study, which was approved by the Local Ethics Committee of the Medical Faculty of the University of Magdeburg in compliance with national legislation and the Code of Ethical Principles for Medical Research Involving Human Subjects of the World Medical Association (Declaration of Helsinki).

### Experimental Paradigm

**FIGURE 3:**
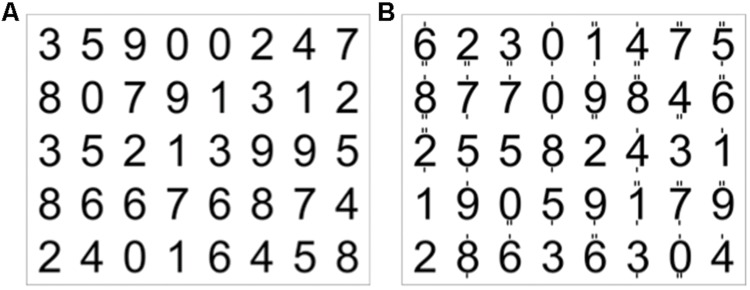
**Example of a 5 × 8 matrix of digits **(A)**, which the subject had to encode within 60 s. (B)** Digits with dashes on top or below. The subjects had to indicate by button press whether or not each digit was even (zero had to be treated as even) and marked by exactly two dashes (targets in the first row were the 2 and the 0, in the second row 0, 8, 4, 6, and in the third row 4).

The experiment was performed using a block design of eight blocks for the *encoding* condition (60 s duration) and eight blocks for the *attention* condition (20 s duration). During the encoding condition a complete 5 × 8 matrix of 40 digits was presented visually, and the subjects were asked to memorize as many items as possible (first row from left, then second row, etc.). This *encoding* condition was followed by a 20-s *attention* condition, where a matrix with different digits was visually presented (**Figure 3**). Here, the subjects had to indicate, by pressing one of two buttons, whether or not each digit was even and was marked by exactly two dashes when adding up the dashes below and above the digit. Zero had to be treated as an even number. The attention condition served to stop the encoding process, and thus controlled for both the visual input and attentional load.

**FIGURE 4:**
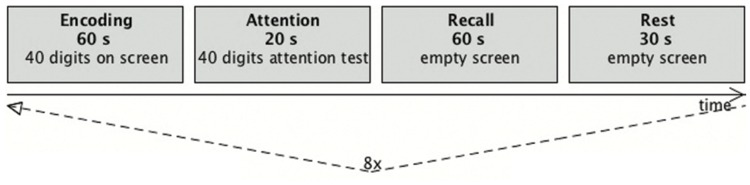
**Diagram of the trial scheme.** One run consisted of a set of four blocks (encoding, attention, recall, and rest). During the recall and rest condition a fixation cross was presented. After an initial rest phase of 30 s each run was repeated eight times, resulting in 695 data sets.

These first two blocks were followed by a recall condition (60 s duration) in which the subjects had to recall as many digits as possible and to say the numbers aloud in the same order as they where asked to memorize them (**Figure 4**). The subjects were instructed to not move their head while talking. The sounds were recorded with a dual channel microphone (MR-Confon, Germany) that subtracted the scanner noise, which enabled the experimental supervisor in the monitor room to easily identify the spoken numbers. The recall condition was followed by a 30-s rest condition. Here, the subjects had to recite the alphabet in silence before the next block was started. The visual stimuli were presented by a video projector onto a back-projection screen, which was viewed from inside the scanner through a mirror system. For stimulus presentation and recording of the behavioral responses, the software application *Presentation* (Neurobehavioral Systems, Albany, NY, USA) was used.

### Data Acquisition and Data Analysis

Magnetic resonance imaging was carried out using a 3 T Trio MRI scanner (Siemens Medical Solutions) equipped with an eight-channel phased array coil. Anatomical data of the whole brain were acquired with high resolution using a multiplanar rapidly acquired gradient echo (MPRAGE) sequence with 1.0 mm^3^ isotropic resolution. For later alignment with the anatomical data an additional inversion-recovery echo-planar imaging (IR-EPI) was acquired prior to each functional run. The IR-EPI exhibited the same geometry as the subsequent functional measurements. 695 volumes were acquired in 23 h 10 min using a gradient-recalled echo-planar imaging (EPI) pulse sequence [echo time (TE) = 30 ms; repetition time (TR) = 2000 ms; flip angle (FA) = 80°; matrix 64 × 64; field of view (FOV) = 19.2 cm; 33 slices of 3 mm, 0.3 mm gap).

The functional data were analyzed with the software system BrainVoyager^TM^QX (Brain Innovation, Maastricht, Netherlands). A standard chain of pre-processing steps, slice scan time correction, 3D-motion correction, linear trend removal, filtering with a high-pass of three cycles per scan, and spatial smoothing with a Gaussian filter with 4 mm full width at half maximum was applied. The functional data were projected onto the corresponding IR-EPI images, co-registered with the 3D dataset, and then transformed to Talairach space.

Due to the low number of subjects, a fixed effects analysis of the group data was performed using a conservative significance level of *p* < 0.01 (Bonferroni corrected) with a minimum cluster size of eight voxels. To determine brain regions exhibiting increased activation under specific conditions in SMs as compared to in control subjects, a conjunction analysis was performed that ensures that the resulting brain regions show a positive deflection of the BOLD response during encoding or recall vs. rest and is controlled for general attentional processes (comparison vs. attention condition). Thus, the contrast for the encoding-specific activation in SMs was: *Encode* in SMs vs. *Rest* in SMs AND *Encode* in SMs vs. *Attend* in SMs AND *Encode* in SMs vs. *Encode* in controls. The contrast for the recall-specific activation in SMs was: *Recall* in SMs vs. *Rest* in SMs AND *Recall* in SMs vs. *Attend* in SMs AND *Recall* in SMs vs. *Recall* in controls.

This analysis is rather conservative but it guarantees that the BOLD response during encoding/recall in SMs is significantly stronger. Beside the comparison between encoding in SMs and encoding in controls and the same for recall, two additional conditions must be fulfilled. Firstly, the activation of the SMs during the encoding/recall phases must be significantly stronger than during the attentional control condition of the SMs because we do not want to discuss brain areas which are strongly activated due to general attention differences between the SMs and the control group. Secondly, we only want to consider brain areas that show a positive BOLD response compared to a resting condition in which the SMs only need to recite the alphabet. This is especially important for the identification of areas involved in the recall where overt reciting of numbers is required by the memorizers. All three contrasts must be independently significant at the *p* < 0.01 level.

## Results

### Task Performance and fMRI Data

For the behavioral analysis we calculated the mean values and standard errors of the mean (SEM). On average, the SMs were capable to recall 34.5 of the 40 digits (*SEM* = 2.37), while the naïve subjects recalled only 10.8 digits (*SEM* = 1.58). The SMs recalled significantly more digits then the controls (*t*-test: *p* < 0.0001, *t* = 7.6, and effect size: *d* > 4). The mean time for recalling all numbers was 37.1 s (1.2 s/digit; *SEM* = 0.15 s) for SMs, and 24.3 s (2.5 s/digit; *SEM* = 0.41 s) for the naïve subjects. The SMs recalled the digits significantly faster (*t*-test: *p* < 0.01, *t* = 2.86, and effect size: *d* > 1.5). The difference in overall duration was due to fewer digits memorized by the control group, as well as to a high variation in recall speed. In some cases the number of recalled digits was too small to fill the entire recall interval. In other cases the naïve subjects recalled the numbers so slowly that the duration of the recall phase was used up completely.

**FIGURE 5:**
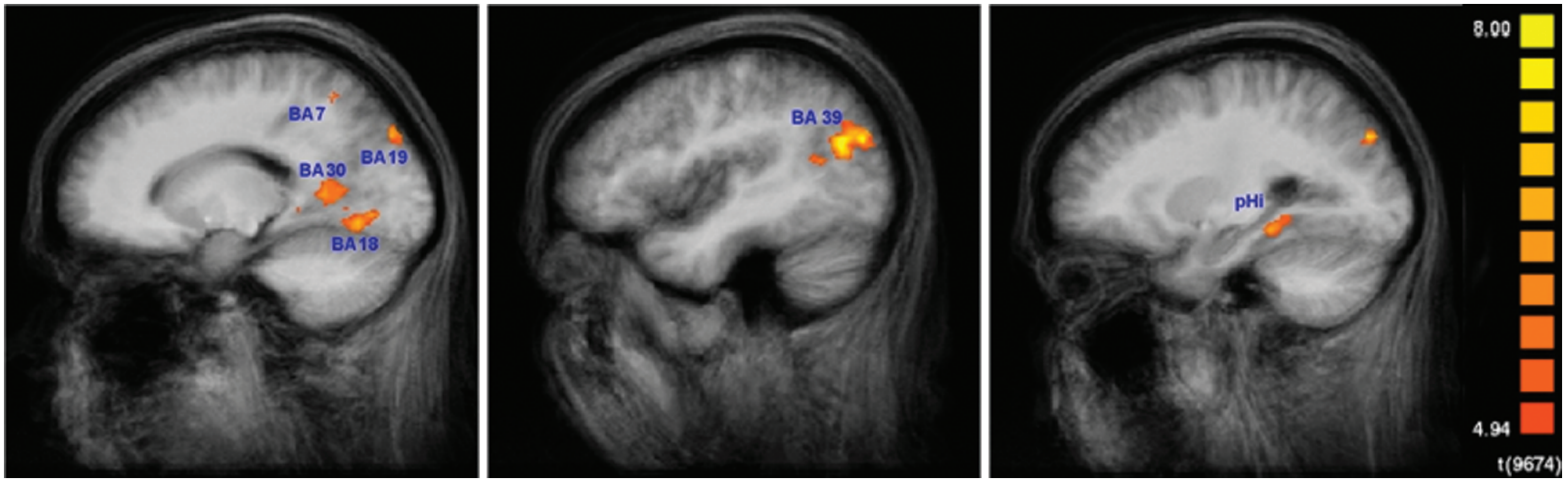
**Encoding-specific activation in superior memorizers.** Contrast: *Encode* in superior memorizers vs. *Rest* in superior memorizers AND *Encode* in superior memorizers vs. *Attend* in superior memorizers AND *Encode* in superior memorizers vs. *Encode* in controls. Color-encoded activation maps overlaid on group-averaged anatomical images (*p* < 0.01, Bonferroni corrected, minimum cluster size 8 voxel) (left *x* = -16, middle *x* = -42, and right *x* = -25).

**FIGURE 6:**
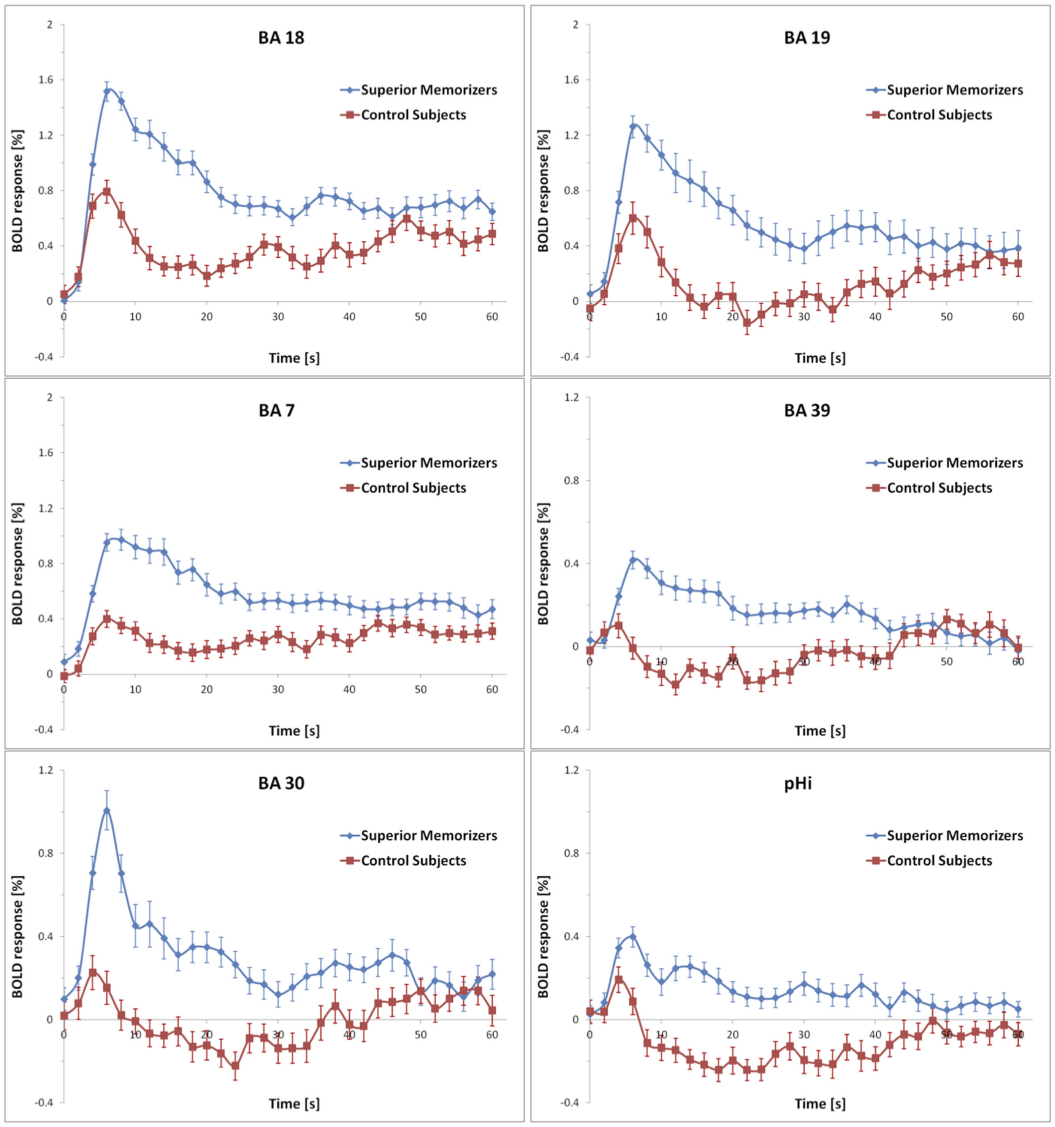
**Averaged BOLD responses of the brain areas showing encoding-specific activation in superior memorizers.** Time point zero denotes the start of the encoding condition that lasted for 60 s. The baseline (0 % BOLD response) was defined as the average response within 4 s prior to the encoding condition. Note the different scaling of the ordinates. The BOLD response of control subjects is depicted for comparison.

**Figure 5** shows the results for the conjunction analysis used to detect brain regions that were significantly more activated during the encoding process in SMs than in naïve subjects. It shows that a network of left hemisphere regions is involved in encoding, consisting of left secondary visual areas (BA 18, 19), left medial superior parietal cortex (BA 7), and left middle temporal gyrus (BA 39). The bilaterally activated retrosplenial cortex (BA 30), and posterior parahippocampal gyrus (pHi) also exhibited a very strong lateralization to the left hemisphere. The details are summarized in **Table 1**. To demonstrate the extent of the differences in brain activation between both groups, the time course of the BOLD response during the 60 s of encoding is displayed in **Figure 6** for each brain area, which was found to be significantly stronger in SMs than in naïve control subjects.

**Table 1:** Regions activated to a higher degree in superior memorizers than in naïve controls (*p* < 0.01, Bonferroni corrected, minimum cluster size eight voxels).

Region	Talairach coordinates			BA	Cluster size
	*x*	*y*	*z*		
**Encoding**				
Left visual cortex	-13	-67	-3	18	1213
Right visual cortex	10	-72	-2	18	228
Left visual cortex	-20	-81	34	19	615
Left angular gyrus	-40	-65	24	39	2162
Left retrosplenial cortex	-12	-53	8	30	3914
Right retrosplenial cortex	13	-51	8	30	1047
Left superior parietal cortex	-20	-57	52	7	263
Left parahippocampus	-25	-36	-7		449
**Recall**				
Left anterior superior temporal gyrus	-57	-8	7	22	346
Right motor cortex	50	-8	30	4/6	676

**FIGURE 7:**
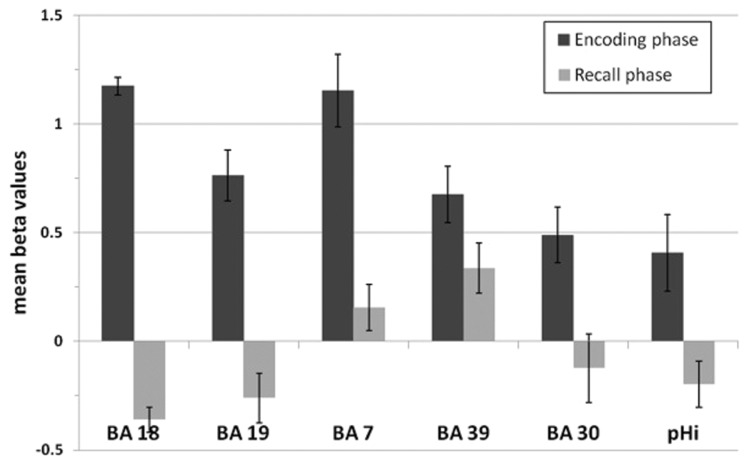
**Mean beta values of the encoding-specific areas of superior memorizers shown in Figure 5.** Beta values of the encoding phase correspond to the BOLD response of the superior memorizers depicted as blue curve in **Figure 6**. The beta values of the recall phase are all significantly lower than those of the encoding phase (BA 18, BA 19, BA 7, BA 30, and pHi: *p* < 0.01; BA 39: *p* < 0.05; two sides *t*-test).

**FIGURE 8:**
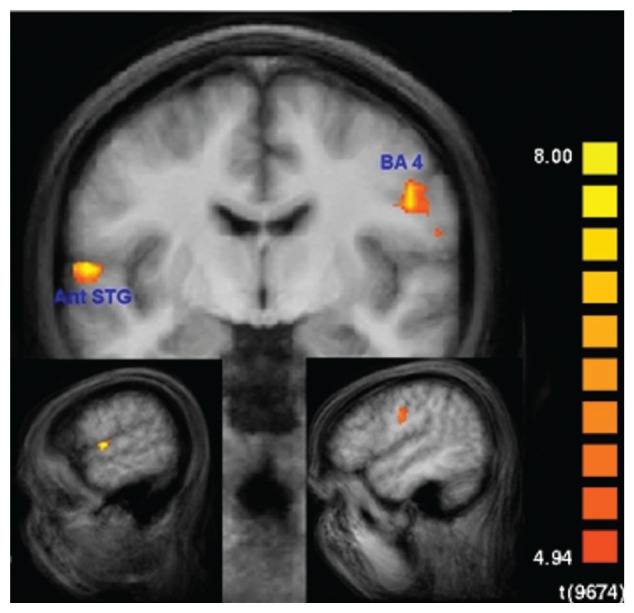
**Recall-specific activation in superior memorizers.** Left anterior superior temporal gyrus (STG) and right motor cortex are the only regions that are specifically activated during the recall condition in superior memorizers. Contrast: *Recall* in superior memorizers vs. *Rest* in superior memorizers AND *Recall* in superior memorizers vs. *Attend* in superior memorizers AND *Recall* in superior memorizers vs. *Recall* in controls. (*p* < 0.01, Bonferroni corrected, minimum cluster size 8 voxel) (down left *x* = -56, down right *x* = 48, and middle *y* = -8).

**FIGURE 9:**
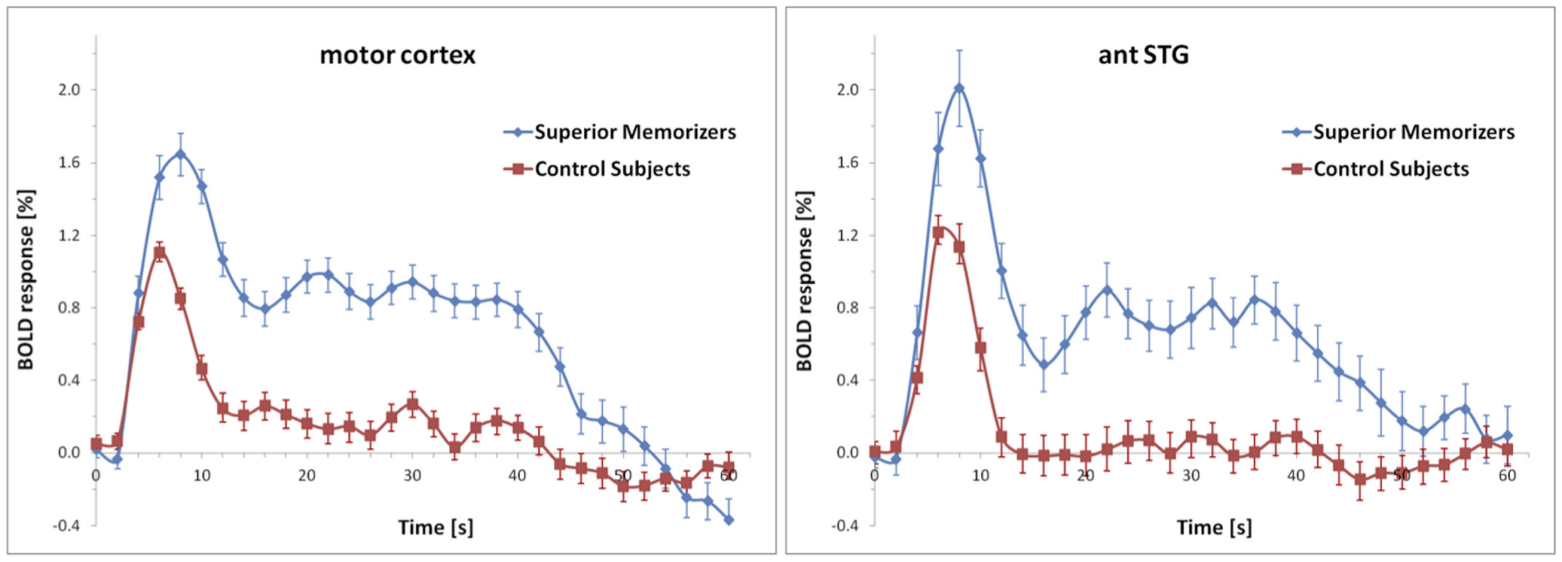
**Averaged BOLD responses of the brain areas depicted in **Figure 8** showing recall-specific activation in superior memorizers.** Time point zero denotes the start of the recall condition that lasted for 60 s. The baseline (0% BOLD response) was defined as the average response within four seconds prior to the recall condition. The BOLD response of control subjects is depicted for comparison.

None of these regions was significantly activated in the recall condition of the SMs. **Figure 7** shows that the beta values of the recall condition were mostly negative and all significantly lower than those of the encoding condition (**Figure 7**). Instead, the left anterior superior temporal gyrus (STG) and the right motor cortex were specifically activated (**Figure 8**). The BOLD response during the 60 s when recalling the digits is displayed in **Figure 9**.

## Discussion

We now discuss the activated brain regions with respect to the involved processes including information transformation and binding processes of the suggested mnemonic procedure (**Figure 1**). The encoding procedure includes the activation of long-term memory, which was found to be characteristic of studies when experts where involved (Guida et al., 2012). This results in mental visualization of a location on the previously memorized internal route. This explains the strong activation in visual brain regions (Johnson and Johnson, 2014). Other contributions to the activation of the visual cortex may come from several sub-processes of translating the digits into visual objects, i.e., in-depth visual perception of digits, recall of the specific letters representing these digits from the previously memorized table, and the search for a correct word containing the letters. Furthermore, several studies also suggest that BA 18 is involved in recollection processes (Spaniol et al., 2009). The fact that SMs use contextual information while applying the MoL supports these findings. Furthermore the use of long-term memory.

The left lateralized effect in BA 19 suggests the involvement of the language-related system when transforming digits into letters and subsequently into words, since a left lateralization of BA 19 was often found to play a role in phonological processing and forming words (Dietz et al., 2005), as well as in word recognition (Cabeza and Nyberg, 2000). One step of the proposed model in **Figure 3** involves silently speaking. However, we did not find specific activation in, e.g., Broca’s area. One explanation is that the control subjects also used inner speech to encode as many digits as possible. Moreover, because the SMs in the group were highly experienced in the encoding process, some of the sub-processes involving covert speech may have required less mental effort.

When connecting the mentally visualized object to the selected landmark, the visualized object becomes navigationally relevant. This may explain why the pHi is activated (Wegman and Janzen, 2011). Moreover, the repetition of the processes depicted in (**Figure 1**) automatically results in mental navigation that is known to involve not only the pHi but also BA 7 and 30 (Ghaem et al., 1997; Maguire et al., 1998, 2003; Kondo et al., 2005; Epstein, 2008; Auger et al., 2012). With the left-hemispheric activation of BA 7 and BA 39 during encoding, we found two areas that have been also found activated by other groups during recollection (Vilberg and Rugg, 2007; Spaniol et al., 2009). Those findings imply that the encoding process of SMs is basically a mixture of encoding and retrieval, as suggested by the scheme of **Figure 1**, where it is shown that items already learned may be used to memorize other new items.

During recall none of the encoding-related areas was specifically activated and the overall number of activated voxels was much smaller than in the encoding condition. This was surprising, since a comparison of **Figures 1** and **2** leads one to expect to find at least the same areas activated for mental navigation as for encoding. This result seems to stand in some contradiction to the transfer-appropriate process theory, which proposes that successful retrieval is accompanied by neuronal processes similar to those occurring during encoding (Rugg et al., 2008). However such a conclusion must be considered very carefully since due to our conjunction analyses we only analyzed very specific activated brain regions.

During recall, the activation in the motor area may be explained by the fact that SMs had to recite significantly more digits. This is in part supported by the BOLD response (**Figure 9**) which in control subjects falls off to baseline much earlier than in SMs. The anterior temporal lobe has been shown to be involved in the retrieval of proper names (Damasio et al., 2004; Bethmann et al., 2012), which are composed of more semantic features than common objects. The left anterior temporal lobe has also been shown to be involved in syntactic processing, independent of syntactic complexity (Friederici, 2002; Dronkers et al., 2004). Hence, it has been suggested that the left anterior temporal lobe contributes to the composition of sentence meaning (Vandenberghe et al., 2002; Humphries et al., 2006). Beyond that, the anterior temporal region has been proposed to be involved in the concatenation of sentences or propositions into stories (Mar, 2004).

Taking these suggestions together, we interpret the current recall-specific activation in the anterior STG to be related to the reconstruction of the exact sequence of objects along the mental route of landmarks. Because SMs make up vivid stories of how the visual objects, which represent the encoded information, are related to the landmarks of their routes, recalling the sequence of encoded information may be interpreted as reviewing a story rather than as mental navigation along a route. Such a difference in strategy may also explain the unexpected difference in the recruitment of brain areas during the encoding compared with the recall phase. Although we think that the described process in **Figure 2** is very accurate, it has to be considered that some modifications would be appropriate. Our results show that the process of mental movement from one location to the next seems to be much less intense than during encoding. Thus it may be reasonable to link directly from the last step of the process, “recall digits related to keyword”, to the step “previously created story pops up”. We have shown that with the dashed arrow in **Figure 2**.

Although many aspects of memory-related processes have been investigated, little is known about persons who have trained their capabilities to memorize abstract and emotionally neutral data using the MoL (Maguire et al., 2003; Kondo et al., 2005; Konrad, 2014). The regions activated during encoding are consistent with those found by Maguire et al. (2003), except that we found activation in the pHi instead of in the hippocampus. The former was also found to be strongly activated in several other encoding studies (Spaniol et al., 2009). One explanation for the higher activation in SMs could be the fact that they encode additional information (e.g., the connection between landmark and object), since the pHi is also suggested by other studies (Epstein, 2008) to be involved in the encoding of contextual information (Davachi, 2006; Charan Ranganath, 2012) and navigationally relevant object information (Epstein, 2008; Wegman and Janzen, 2011). In another study naïve subjects were trained to use the MoL prior to the fMRI experiment (Kondo et al., 2005), which resulted in activation of the left fusiform and lingual gyrus during both encoding and recall. However, it should be noted that the subjects in this study were trained only briefly and thus were by no means SMs.

According to the hemispheric encoding/retrieval asymmetry model (HERA; Tulving et al., 1994) the left PFC should be more involved during encoding and the right PFC more involved during retrieval. We found a strong left lateralization for a few areas (**Table 1**) specifically activated by SMs during encoding but no specific activation in the PFC during encoding or during retrieval. The overall left dominance could indicate language-related and sequential processing while applying the MoL combined with a phonetic system.

In this study we aimed to interpret the fMRI data of SMs who used the MoL combined with a phonetic system to memorize long lists of digits. To understand which neuronal processes may potentially be involved, a scheme was developed of the different sub-processes that are employed when SMs fulfill this task. Although our data show some support for the proposition that language-related systems are involved in addition to the brain parts already known to play a role in encoding, this interpretation of the data is limited at the moment by the fact that the single sub-processes of the scheme cannot be separated within our experimental setup. Furthermore the overt behavior during the different conditions was different, which may cause some background noise in the data and some lack of control over all potential sub-processes. Thus there is a need for future studies focusing on partial aspects of the overall process. This could be achieved by scanning the SMs solely while they “walk” through their mental route without creating mental images or while they translate numbers into images without connecting them to specific locations.

A limitation of the fixed effects analysis is that it does not allow for inferences to the population. Nevertheless, to demonstrate the significance of the effects, we used very conservative criteria, as described in the Section “Materials and Methods”. Furthermore, we show the BOLD response over time for each activated brain area that reveals how large the difference between SMs and controls is in terms of BOLD response amplitude. It also has to be stated that the potential heterogeneity of strategies of the naïve volunteers may limit the generalization of the results, which makes more specific research necessary.

In sum, we found that SMs use different networks for encoding and recall, with the latter process generating much less brain activation. This is in accordance with the everyday challenges of learning a language, playing a piece of music, or learning movement patterns. Once a pattern is learned it can be recalled and executed very quickly and with little conscious effort. However, learning strategies are seldom taught in schools or other institutions. One may speculate to which degree learning would gain efficiency when applying optimized strategies instead of trying to memorize data by endless repetition. At least our behavioral results show that the strategies of the superior memorizer are very efficient for learning digits. It would be interesting to investigate the efficiency of their strategies on general knowledge. Such a study could have an important influence on how knowledge is imparted in schools or universities.

## Author Contributions

JM, ABr, and JB wrote the main manuscript text. JM also prepared **Figures 1–4**. ABr prepared **Figures 5–9**, was responsible for methods and data analysis. ABr, ABe, and ML contributed to the discussion. All authors reviewed the manuscript.

## Conflict of Interest Statement

The authors declare that the research was conducted in the absence of any commercial or financial relationships that could be construed as a potential conflict of interest.
